# Demirjian’s and Cameriere’s Methods for the Assessment of Dental Age Estimation in Children from a Southern Brazilian City

**DOI:** 10.3390/diagnostics14141513

**Published:** 2024-07-13

**Authors:** Julia Carelli, Gabriela Sabrina da Silva, Mariana Vegini Gomes, Thais Vilalba, Flares Baratto-Filho, João Armando Brancher, Svenja Beisel-Memmert, Christian Kirschneck, Celia Maria Condeixa de França Lopes, Alexandre Moro, Erika Calvano Küchler

**Affiliations:** 1 School of Dentistry, University of Joinville, Joinville 89219-710, Brazil; jucarelli_@hotmail.com (J.C.); gabrielasabrina.s@hotmail.com (G.S.d.S.); gomesvmariana@gmail.com (M.V.G.); fbaratto1@gmail.com (F.B.-F.); cmcflopes@gmail.com (C.M.C.d.F.L.); 2 Department of Orthodontics, School of Dentistry, Positivo University, Curitiba 82010-330, Brazil; alexandremoro@uol.com.br; 3 School of Dentistry, Tuiuti University of Paraná, Curitiba 82010-330, Brazil; thaisvilalba@gmail.com; 4 School of Anatomy, Federal University of Paraná, Curitiba 82010-330, Brazil; brancher.a@gmail.com; 5 Department of Orthodontics, University Hospital Bonn, Medical Faculty, Welschnonnenstr. 17, 53111 Bonn, Germany; svenja.memmert@ukbonn.de (S.B.-M.); christian.kirschneck@uni-bonn.de (C.K.)

**Keywords:** panoramic radiography, odontogenesis, orthodontics, dental age, chronological age

## Abstract

The chronological age estimation of living individuals is a crucial part of forensic practice and clinical practice, such as in orthodontic treatment. It is well-known that methods for age estimation in living children should be tested on different populations. Ethnic affiliations in Brazil are divided into several major groups depending on the region, with the south of Brazil being known for its German immigration. (1) Background: This study aimed to evaluate the correlation between chronological age and dental age using Demirjian’s method and Cameriere’s method in a group of children from Joinville, South Brazil to investigate if both methods can be used to estimate dental age in this population. (2) Methods: The sample consisted of 229 panoramic radiographs (119 were males and were 110 females) from Brazilian children (ages ranging from 6 to 12 years). The chronological age at the time of the panoramic radiographic exam was calculated for each child. The dental age was estimated according to Demirjian’s method and Cameriere’s method. All continuous data were tested for normality by using the Shapiro–Wilk test. The Pearson correlation coefficient test was applied. An alpha of 5% (*p* < 0.05) was used for all analyses. (3) Results: The mean chronological age was 8.75 years. According to Demirjian’s method, the mean dental age was 9.3 years, while according to Cameriere’s method, the mean dental age was 8.66 years. A strong correlation between chronological age and dental age according to Demirjian (r = 0.776 and *p* < 0.0001) and Cameriere (r = 0.735 and *p* < 0.0001) was observed for both genders. (4) Conclusions: Both methods presented a good correlation with chronological age in the studied population and could be used to assess dental age in this population.

## 1. Introduction

To assess chronological age using parts of the human body, performing bone analysis is an essential tool to identify individuals’ age and aid in solving crimes [1]. The bone age of a child indicates his or her level of biological and structural maturity. The analysis of anatomical regions in the hand and wrist area is the most common modality for skeletal age determination in clinical practice [2]. However, other skeletal structures, such as the analysis of cervical vertebrae in cephalometric radiographs, also present high-accuracy methods. Tooth structures also follow a predictable pattern of development, mineralization, and eruption in the oral cavity during odontogenesis. Therefore, the estimation of chronological age based on dental development stages may also be an accurate, useful, and simple method [3,4]. In fact, some evidence in the current literature supports that dental age determination by stage of dental maturation analysis could be a more reliable indicator of chronological age than skeletal analysis because dental development is less affected by various conditions. The growth and development of the human skeleton require an adequate supply of many different nutritional factors and are also affected by environmental factors, systemic diseases, and hormonal conditions [5,6]. Therefore, dental age assessment (using different methods) is one of the most reliable techniques of chronological age estimation used for criminal, forensic, and anthropologic purposes.

Several dental age determination methods have been proposed in the literature in recent decades [1], and the most commonly used in different populations is Demirjian’s method from 1973. Demirjian’s method of dental age assessment estimates the overall age by scoring based on the stage of teeth formation, using dental images from panoramic radiographs or cone beam computed tomography (CBCT). Panoramic radiographs include dental X-rays of the upper (maxilla) and lower jaw (mandible). These radiographs show a two-dimensional (2D) view of a half-circle from ear to ear. CBCT, on the other hand, consists of three-dimensional (3D) images of hard and soft tissue structures. Demirjian’s method was primarily based on data acquired from individuals of French-Canadian origin. This method has been modified and adapted over the years to improve its accuracy and applicability for chronological age estimation in panoramic radiographs [2]. There is high evidence that CBCT scans also be used to obtain reproducible and reliable dental age estimations [7]. 

Demirjian’s method is widely used, and numerous studies have been performed utilizing this method in different ethnic populations. It is commonly considered in the literature as the gold standard. This method assesses the mineralization stages of seven permanent teeth on the left side of the mandible (from the central incisor to the second molar, whether erupted or not) using dental panoramic radiographs. The calcification of a tooth is divided into eight stages, and each stage has a designated score that is different for boys and girls. The results obtained are then compared with an established age estimation table for the final result per child [2].

More recently, a new method to assess dental age was introduced by Cameriere in 2006 [8]. This method also uses dental panoramic radiographs. The Cameriere method analyzes dental maturation by measuring the projections of open dental apices through a formula that analyzes tooth maturation by estimating open apices of seven permanent teeth on the left mandible of the radiograph [8]. This method is also a widely accepted approach to age estimation in children.

Dental age is an important biological age marker that plays a role in many fields, including forensic science, anthropology, pediatrics, orthodontics, and pediatric dentistry [9]. Despite both Demirjian’s method and Cameriere’s method showing reliability and accuracy in age estimation, there is still certain controversy in the literature, especially when applied to different populations [1]. Therefore, new studies in different samples are important to identify the best method to determine the dental age in different ethnic groups. 

Brazil’s ethnicity is distributed among several major groups depending on the region. One interesting fact in the human geography of the south of Brazil is that the three southern states include a significant proportion of German descendants. German immigration allowed the formation of the German-Brazilian ethnicity [10]. Several German colonies were started in northeast Santa Catarina. One well-planned colony in which the Germans settled was Joinville. Nowadays, Joinville is the largest city in Santa Catarina and is well-known for its German immigration origin. Thus, in the current study, we investigate this specific population (from Joinville, South Brazil) in order to analyze the performance of two dental age estimation methods in children from Joinville. Our study evaluated the correlation between chronological age and dental age using Demirjian’s method and Cameriere’s method to investigate if both methods can be used to estimate dental age in this population.

## 2. Materials and Methods

### 2.1. Ethical Aspects, Study Design and Sampling

This retrospective observational study was approved by the Research Ethics Committee of the University of Joinville—Santa Catarina(UNIVILLE) (under the approval number: 4.392.279 in 11 September 2020). The study was conducted in accordance with the Declaration of Helsinki (Ethical Principles for Medical Research), revised in 2013. The study population was a random sample, i.e., a non-probability sample. Dental records of all children who received dental treatment between March 2021 and November 2023 in the city of Joinville at the Faculty of Dentistry of the University of Joinville (Pediatric Dental Clinic) were examined. The children’s ages ranged from 6 to 12 years. All included children were residents of the Joinville region. Joinville is the largest city in Santa Catarina state, which is located in the Southern Region of Brazil (latitude and longitude coordinates are −26.304516, −48.843380). Joinville is a center of industrial area, with a very multinational and multicultural community and has around 605,000 inhabitants, most of whom are of German descent. From 1851 to 1888, the city of Joinville attracted thousands of German immigrants to the region [11].

The inclusion criteria were healthy patients living in Joinville city or in the region and children no older than 13 years. The exclusion criteria were children who did not have a panoramic radiograph in their clinical records, or who had a panoramic radiograph with image quality problems affecting permanent tooth visualization. Furthermore, children with systemic diseases, cleft lip and or palate, facial trauma, previous history of facial surgery, dental extraction of permanent tooth/teeth, and dental agenesis were excluded. The dental records of the children were screened and the data were collected between November 2021 and August 2022.

### 2.2. Dental Age Determination

The dental record was assessed to analyze the date of birth and the gender (male or female) reported in the anamnesis by the caregivers. The chronological age was then calculated for each child by subtracting the date of birth from the date of the imaging exam (the day that the panoramic radiographic was performed), and the results were expressed as years with two decimal places. For the evaluation of dental age, the chronological age of the participants was blinded during the analysis. The intra-rater and inter-rater reliability was examined using weighted kappa statistics after the images of 5 randomly selected panoramic radiographs of the participants were analyzed. The radiographs were evaluated twice in a blinded manner, both by the same investigator as well as by a second (senior) investigator with a two-week interval. The intra-rater and inter-rater reliability were determined by calculating Cohen’s kappa. Cohen’s kappa was good to excellent (intra-rater was k = 1.00 and inter-rater k = 0.86).

The dental age was estimated according to Demirjian’s method and Cameriere’s method. The dental age estimation according to Demirjian’s method was performed based on the maturity of the seven permanent teeth on the lower left side (mandible) (except the third molar, which is not included in this analysis). According to Demirjian’s staging criteria [2], each tooth was classified into 8 different developmental mineralization stages, which were identified by the letters “A” to “H”. The designated stages started with the initial dental crown formation and continued until the closure of the root apex. Each tooth was rated on a scale, and each rating was then converted into a score. The description of the calcification stages is as follows:

(A) In both uniradicular teeth and multiradicular teeth, the beginning of calcification is observed at the superior level of the crypt in the form of an inverted cone or cones, without the fusion of these calcification points.
(B) The fusion of calcified points forms one or more cusps, which bond to give a regularly outlined occlusal surface, or mineralized cusps are joint so the mature coronal morphology is well defined.
(C) The dental crown is half-formed, the pulp chamber is evident, and dentinal deposition is taking place.
(D) The dental crown formation of uniradicular teeth and multiradicular teeth is completed down to the cementoenamel junction, the pulp chamber has a trapezoidal form, and the beginning of dental root formation is observed.
(E) Initial formation of the radicular bifurcation is observed, and the dental root length is still less than the dental crown height.
(F) The apex ends in a funnel shape; the root length is equal to or greater than the crown height.
(G) The walls of the root canal are now parallel, and its apical end is still partially open.
(H) The apical end of the dental root canal is completely closed; the periodontal membrane has a uniform width around the dental root and the apex.

Cameriere’s method [8] analyzed the left permanent mandibular teeth, and the age was calculated using the following formula: Age = 8.971 + 0.375g + 1.631 × 5 + 0.674N0 − 1.034s − 0.176sN0 where g is a variable equal to 1 for boys and 0 for girls. The total number of teeth with completely closed apices (N0) was obtained. For teeth with a single root, the width of the open apex was measured (A1–A5). For teeth with two roots, the sum of the widths of the two open apices was measured (A5–A7). The measurements (A) were normalized by dividing the tooth length (L1–L7) to consider the effect of possible differences in magnification and angulations among radiographs. The normalized measurements of the seven left permanent mandibular teeth (xi = Ai/Li, i = 1… 7), the sum of the normalized open apices (s), and the number of teeth with closed apices (N0) were used to estimate dental age.

**Figure 1:**
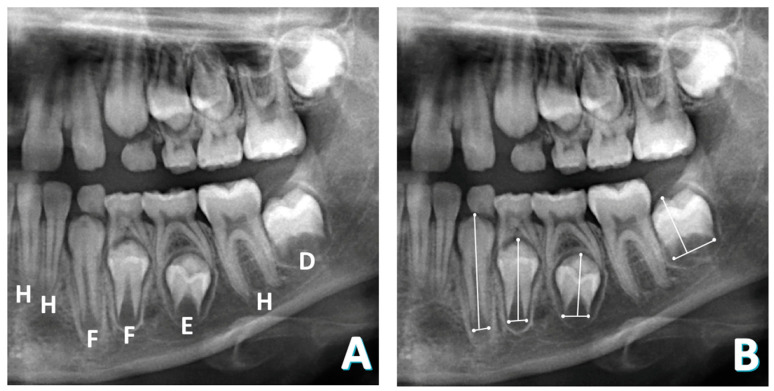
Dental age determination methods in panoramic radiograph. (**A**) Demirjian’s method; (**B**) Cameriere’s method.

Both methods are demonstrated in Figure 1.

### 2.3. Statistical Analysis

The data from all the included children were tabulated in an Excel sheet (Excel 2017 Microsoft Office). All statistical analyses were performed using the software Graph Pad Prism 5.0a (Graph Pad Software Inc., San Diego, CA, USA). All continuous data were tested for normality by the Shapiro–Wilk test. 

The intra-rater and inter-rater reliability were assessed by calculating Cohen’s kappa (k = 1.00 and k = 0.86, respectively).

The Pearson correlation coefficient test was used to determine the correlation strength between the variables. The strength of the positive correlations was defined according to the value of the “Correlation Coefficient”, such as 1: perfect correlation; 0.7 to 0.9: strong correlation; 0.4 to 0.6: moderate correlation; 0.1 to 0.3: weak correlation; and 0: no correlation. 

The analyses were performed and described in the total sample and stratified according to gender (male and female). The statistical significance was defined as two-tailed with an adopted alpha established in 5% for all comparisons performed (*p* < 0.05).

A post-hoc power analysis for the results reported in this article was conducted. 

## 3. Results

A total of 9117 dental records were screened for eligibility in the pediatric dental clinic of the UNILLE dental school. Thus, 8231 dental records were excluded for the absence of dental panoramic radiographs. In the second step of the project’s screening process, 657 dental records were excluded due to poor image quality associated or not with lower permanent tooth visualization, systemic diseases, dental agenesis, or cleft lip and/or palate. Finally, a total of 229 (119 males and 110 females) dental records were included in this study. In the case of patients with multiple dental panoramic radiographs, only one radiograph per child was analyzed, and the chronological age was calculated according to the included radiograph.

The median chronological age of the included sample at the time of the radiograph exam was 8.75 years (ranging from 6 to 12.08). According to Demirjian’s method, the median dental age was 9.3 years (ranging from 6.7 to 13.40), while according to Cameriere’s method, the median dental age was 8.66 years (ranging from 5.63 to 11.18). 

These descriptive characteristics of the children (both sexes) included in the study are presented in Table 1.

Table 2 presents the descriptive statistics regarding the chronological and dental age in both methods according to sex (male and female).

The results of Pearson’s correlation analysis between dental age according to Demirjian’s method and Cameriere’s method with chronological age (in both sexes) are shown in Table 3.

A strong correlation was observed for all analyses, which ranged from r = 0.725 (in Cameriere’s method for females) to r = 0.801 (in Demirjian’s method for males), with a statistical association (*p* < 0.0001) for all comparisons. 

The post-hoc power calculation found that the study had 99% power at an alpha level of 5% to detect a significant difference in mean dental age between Demirjian’s method and Cameriere’s method. 

## 4. Discussion

The present study aimed to investigate the association between dental age and chronological age in a group of children of both sexes from a city in the south of Brazil. One important aspect to be highlighted is that a recent study aimed to assess the validity of age estimation methods based on dental maturity indices and their reproducibility through a meta-analysis. The authors pooled a total of 23 studies and concluded that there is only very low-quality evidence and no recommendation on which dental age method to choose. They recommended that future researchers in this field should be aware of the models in use in their countries and which perform best with their local databases [12]. A systematic review of the literature from 2021 focused only on studies performed on Brazilian teenagers. The authors evaluated original studies on dental age estimation methods applied to Brazilians and concluded that most of the international methods for dental age estimation had optimal performance in Brazilian children; however, it is important to emphasize that none of the studies evaluated children from Joinville, which is a population with specific characteristics [13]. The population from Joinville has some differences from other regions of Brazil. Joinville is well-known for its German immigration origin, which could cause some variation in the dental age due to the particular genetic background of the population. Therefore, in the present study, we used two methods to estimate dental age in this specific population. We used Demirjian’s [2] and Cameriere’s [8] methods. Demirjian’s approach has gained widespread recognition and has become the predominant method for estimating dental age [14].

Joinville city is essentially colonized by a German-Brazilian population, composed of German descendants. We hypothesized that the observed results could be similar to results observed in the German population [15] or other studies with central European samples [16] due to the genetic background of the population. In our study, both methods showed a strong correlation between dental age and chronological age. There are some studies that show that Demirjian’s method is geographically sensitive [17,18], except for some studies that are single-population-oriented [13,19]. Cameriere’s method is considered one of the most geographically stable methods [13,17,20].

A systematic overview of dental methods for age assessment in living individuals reported notable tendencies of overestimation and underestimation in some methods for age estimation, which was especially notable in the case of Demirjian (overestimation) and Cameriere (underestimation) methods. Cameriere’s method leads to underestimation in most evaluated populations (13 out of 14) included in the overview (Vila-Banco). In our sample, Cameriere’s method also underestimates the chronological age, which agrees with most of the published studies; however, some studies using Cameriere’s method also showed age overestimation [13,17,20].

It is important to consider that in previous studies, the age overestimation of Demirjian’s method varies between 4 and 9 months [13,17,18]. In our study, an overestimation was also observed. A meta-analysis previously performed on 28 published articles using the Demirjian method to estimate chronological age in 14,109 children described that most papers reported that the Demirjian method significantly overestimated the chronological age. Their meta-analysis also showed a significantly weighted mean difference between the dental age and the chronological age in both genders [14]. Therefore, our study presents results similar to those observed in the literature.

The analysis of dental age is a crucial aspect of clinical practice, particularly in children and young adolescents, and forensic dentistry [21]. Several methods have been developed for this purpose [1] but approaches that use dental tissues are among the most useful and reliable methods due to their small capacity for change and greater resistance to degradation [22]. Unlike skeletal analysis, dental age analysis is less influenced by extrinsic or intrinsic factors, making this approach a more reliable indicator of chronological age in some situations and aspects [5]. It is also less affected by environmental factors such as nutrition and the individual’s endocrine system variations, therefore it is an effective tool for determining age ante-mortem and post-mortem in children [23]. In order to determine the dental age of the studied sample, we used panoramic radiographs from the University of Joinville archives, therefore no individual was exposed to unnecessary ionizing radiation for the radiographic exam as the panoramic radiograph is part of their treatment plan. The analysis of the panoramic radiographs enables the assessment of the individual’s dental development stages while offering the advantages of low radiation exposure and cost-effectiveness for the patient [24].

Both methods tested in this study presented strong correlations between dental age and chronological age. Nair et al. [25] carried out a study comparing Demirjian’s method [2] and Cameriere’s method [8] in an Indian population composed of children aged 7 to 12 years of both sexes and concluded that Cameriere’s method was the most reliable method for estimating the age in that population. In Europe, Wolf et al. [15] compared the two methods in a German population aged 6 to 14 years of both sexes and found that the Demirjian method presented more adequate results for the estimation of age in the investigated population, similar to our results. Some authors reported that Demirjian’s method may be more relevant for age groups between 12 and 18 years [26,27]; however, our study did not include children this age.

Cameriere’s method was tested by Machado et al. [28] in children of the Southeast of Brazil, aged 6 to 14 years old, and showed a good correlation with chronological age for Brazilian boys and girls, which was also observed in our study. Hostiuc et al. [17] showed that Cameriere’s method is useful for estimating the chronological age, with errors of less than one year, and concluded that Cameriere’s method is sufficiently accurate, at least in the 7 to 14 age range.

Finally, it is important to mention that the development of each individual can be affected by other factors in populations around the world, covering individuals from different cities or regions within the same country [29], especially in a country with Brazilian dimensions. Dental age estimation may require its application to dead and living persons from any population. Our results suggest that both methods are efficient in estimating the chronological age of the population from Joinville, Brazil. It is important to highlight the fact that we investigated a relatively small sample size. Another important limitation that we should highlight here is the fact that only children aged from 6 to 12 years were included. Other studies should investigate dental age estimation methods to evaluate their performance in teenagers from this population. Future studies should use artificial intelligence techniques to improve dental age estimation for criminal, forensic dentistry, and anthropological purposes. Forensic cases at medical examiner offices or clustering the victims in mass disasters are the most common scenarios where dental age estimation is used on the dead; criminal and immigration cases are cases where dental age estimation is often applied to the living. Furthermore, dental age estimation is important for orthodontic diagnosis and treatment. In fact, Demirjian’s method combined with machine learning algorithms started to be used recently [30,31] and is a trend for future studies.

## 5. Conclusions

In conclusion, the results of our study show that Demirjian’s method and Cameriere’s method presented good correlations with the chronological age of children from Joinville, in the South of Brazil. Although our findings support that these methods are good in our sample, we suggest that a future populational study should be performed to confirm our results.

## Figures and Tables

**Table 1:** Descriptive statistics of studied variables in both sexes.

	Total Sample				
	Mean	Standard Deviation	Median	Max–Min	Interquartil Range
Chronological age	8.82	1.29	8.75	12.08–6.0	1.91
Demirjian’s method	9.62	1.52	9.3	13.40–6.7	2.45
Cameriere’s method	8.74	1.29	8.66	11.18–5.63	2.42

**Table 2:** Descriptive statistics of studied variables in males and females.

	Male					Female				
	Mean	Standard Deviation	Median	Max–Min	Interquartil Range	Mean	Standard Deviation	Median	Max–Min	Interquartil Range
Chronological age	8.90	1.38	9.0	11.5–6.0	2.20	8.73	1.18	8.75	12.1–6.1	1.66
Demirjian’s method	9.66	1.52	9.4	12.9–6.7	2.4	9.5	1.52	9.1	13.4–7.1	2.60
Cameriere’s method	8.76	1.33	8.65	10.7–5.6	2.44	8.71	1.26	8.69	11.18–6.1	2.47

**Table 3:** Correlation between dental age and chronological age.

Method		Total Sample (Male + Female)	Male	Female
Demirjian’s method	R	0.776	0.801	0.749
	*p*-value	**<0.0001**	**<0.0001**	**<0.0001**
Cameriere’s method	R	0.735	0.745	0.725
	*p*-value	**<0.0001**	**<0.0001**	**<0.0001**

Notes: Pearson’s correlation was performed. Bold forms mean statistical significance.

## Data Availability

Further inquiries can be directed to the corresponding authors upon request.
